# Low-frequency oscillations reflect aberrant tone restoration during the auditory continuity illusion in schizophrenia

**DOI:** 10.1038/s41598-020-68414-3

**Published:** 2020-07-17

**Authors:** Joseph Wooldridge, Mathis Kaiser, Yadira Roa Romero, Lars Riecke, Julian Keil, Daniel Senkowski

**Affiliations:** 1 Department of Psychiatry and Psychotherapy, Charité – Universitätsmedizin Berlin, St. Hedwig Hospital, Große Hamburger Str. 5-11, 10115 Berlin, Germany; 2 Berlin School of Mind and Brain, Humboldt-Universität zu Berlin, Berlin, Germany; 3 Department of Cognitive Neuroscience, Maastricht University, Maastricht, The Netherlands; 4 Biological Psychology, Christian-Albrechts-University Kiel, Kiel, Germany

**Keywords:** Auditory system, Cognitive neuroscience, Sensory processing

## Abstract

Patients with schizophrenia (ScZ) often show impairments in auditory information processing. These impairments have been related to clinical symptoms, such as auditory hallucinations. Some researchers have hypothesized that aberrant low-frequency oscillations contribute to auditory information processing deficits in ScZ. A paradigm for which modulations in low-frequency oscillations are consistently found in healthy individuals is the auditory continuity illusion (ACI), in which restoration processes lead to a perceptual grouping of tone fragments and a mask, so that a physically interrupted sound is perceived as continuous. We used the ACI paradigm to test the hypothesis that low-frequency oscillations play a role in aberrant auditory information processing in patients with ScZ (N = 23). Compared with healthy control participants we found that patients with ScZ show elevated continuity illusions of interrupted, partially-masked tones. Electroencephalography data demonstrate that this elevated continuity perception is reflected by diminished 3 Hz power. This suggests that reduced low-frequency oscillations relate to elevated restoration processes in ScZ. Our findings support the hypothesis that aberrant low-frequency oscillations contribute to altered perception-related auditory information processing in ScZ.

## Introduction

In noisy environments we are often confronted with various acoustic stimuli, e.g., the ongoing sounds of vehicles in traffic. These sounds are sometimes interrupted by other acoustic input and it can be important to perceptually restore the interrupted sounds. Studies in patients with schizophrenia (ScZ) have shown deficits in auditory information processing. This involves basic stimulus processing, such as sensory gating^1^, but also impairments in the fine-grained processing of changes in pitch, duration or location of tones^2,3^. It is likely that these deficits contribute to clinical symptoms in ScZ^4^, such as auditory hallucinations. Abnormal synchronization of ongoing rhythmic activity—so called neural oscillations—appear to be a central neural mechanism underlying perceptual deficits and clinical symptoms in ScZ^5,6^. Neural oscillations at rest appear to be reduced and instable in ScZ^7–9^. Similarly, studies on stimulus processing within and across various domains have indicated reduced local power and phase synchrony in ScZ, indicating impaired neural synchronization^10–14^. Importantly, previous studies have suggested that aberrant low-frequency oscillatory activity could play a central role in auditory information processing deficits in ScZ^15,16^. Thus, there is robust evidence that aberrant neural oscillations are related to clinical symptoms and stimulus processing deficits in ScZ, which could also be evident in impaired perceptual grouping and—conversely—in increased restoration of interrupted sounds.

An elegant paradigm to examine the restoration of interrupted sounds is the auditory continuity illusion (ACI)^17^. In the case of the ACI, auditory restoration leads to a perceptual grouping of tone fragments and a mask, so that a physically discontinuous sound is perceived as continuous^18^. Auditory continuity illusion paradigms have been reported for a wide range of auditory signals in healthy individuals, including modulated tones^19,20^, music^21^, vowels^22^, and more complex speech stimuli^23^. A behavioural study investigating auditory restoration in ScZ reported decreased levels of auditory restoration in ScZ patients with auditory hallucinations compared with healthy control subjects^24^. To date, the neurophysiology of auditory restoration during the ACI has not been investigated in patients with ScZ.

In healthy individuals, electroencephalography (EEG) studies exploring the neural dynamics of auditory restoration during the ACI have emphasized the role of low-frequency oscillatory activity^20,25–27^. An EEG study in healthy individuals investigating oscillatory activity in the ACI employed 3 Hz amplitude-modulated tones that were either interrupted or continuous^20^. Moreover, during the period of interruption, a noise that was either partially masking the tone (i.e. with a spectral gap in the noise centred around the frequency of the tone), or fully masking it (i.e. with no spectral gap in the noise) was overlaid. Perceptually, the interrupted but fully masked tones elicited the illusory perception of continuity in participants. The authors found increased 3–4 Hz power after the onset of interruptions for discontinuous stimuli when compared with the same time window for physically uninterrupted stimuli. Furthermore, the 3–4 Hz power increase was dampened when interruptions were fully masked as opposed to when they were only partially masked. Finally, the 3–4 Hz power increase was dampened when participants perceived a continuity illusion versus when they perceived an interruption. These findings were replicated, to a large extent, in a recent study, where the authors investigated induced power changes and phase locking in response to 3 Hz amplitude-modulated tones during the interval of an interrupting noise^27^. Again, the authors observed an attenuation of 3 Hz power during continuity illusions in comparison to both continuous tones and veridically perceived interrupted tones. Thus, modulation of low-frequency oscillatory activity appears to subserve auditory restoration.

In this high-density EEG study, we investigated auditory restoration during the ACI in patients with ScZ and healthy control participants. We focused on the analysis of 3 Hz oscillations and explored whether any neural alterations in patients would relate to their clinical symptoms. Given previous findings of attenuated low-frequency oscillations during auditory processing in ScZ^28,29^, we expected perceptually relevant alterations in 3 Hz power during the ACI paradigm in patients with ScZ. Behavioural and EEG data for the control group have been previously described^27^. The present study constitutes an extension of this study with a focus on the examination of differences and commonalities in auditory restoration during the ACI paradigm between patients with ScZ and healthy controls.

## Results

EEG data were recorded from patients with ScZ and matched control participants while they rated their perception of continuous and interrupted tones, which were overlaid with full or notched noise masks. Behavioural data and power of 3 Hz oscillations were subjected to univariate ANOVAs based on a linear mixed effects model with fixed effects of Continuity (interrupted vs. continuous), Mask (full mask vs. notched mask), Group (patients vs. controls), and with Subject as a random effect.

### Behaviour

**Figure 1:**
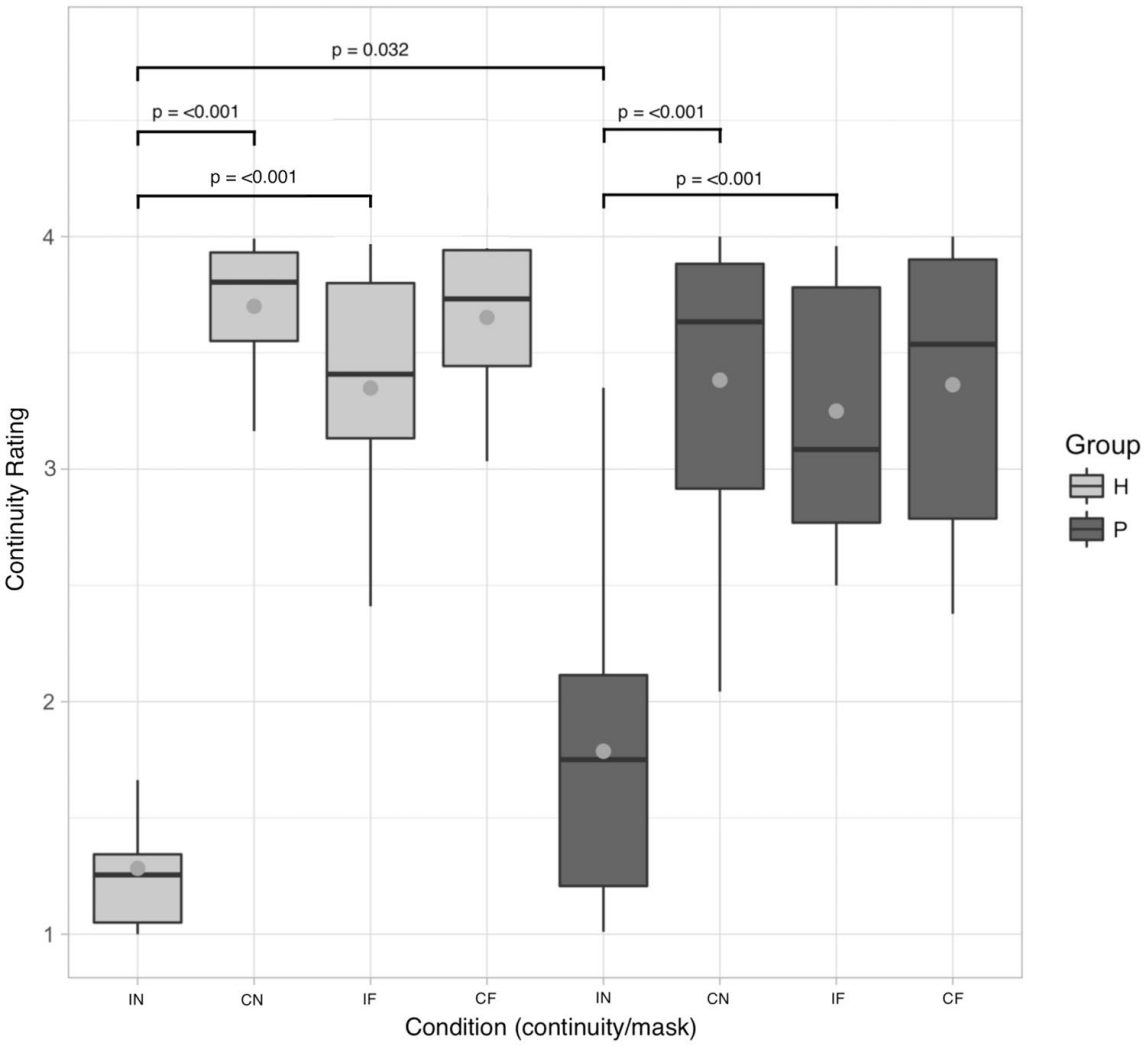
Continuity rating across conditions. Control data (H) is arranged on the left side of the figure (light grey boxes); patient data (P) on the right (dark grey boxes). Grey points indicate mean values. Brackets describe all significant results from planned post-hoc comparisons (see "Analysis of behavioural data" section for details of planned comparisons included in the analysis).

Individuals in both groups reported IF, CN and CF tones on average as continuous, whereas they tended to report IN tones veridically as discontinuous (Fig. 1). Statistical analysis of perceived continuity with Group as a between-group factor revealed main effects for Tone Continuity [*F*(1,105) = 314.61, *p* < 0.001] and Mask [*F*(1,105) = 193.91, *p* < 0.001], but no main effect for Group [*F*(1,35) = 0.14, *p* = 0.704]. However, interactions were found between Tone Continuity and Mask [*F*(1,105) = 209.1, *p* < 0.001], Tone Continuity and Group [*F*(1,105) = 16.92, *p* < 0.001] and additionally across all factors Tone Continuity, Mask and Group [*F*(1, 105) = 6.57, *p* = 0.012]. Follow-up comparisons revealed that the control group rated IF tones as more continuous than IN tones (*z* = 16.15, *p* < 0.001) and IN tones as less continuous than CN tones (*z* = −18.9, *p* < 0.001). Patients showed a similar pattern, also rating IF tones as more continuous than IN tones (*z* = 12.41, *p* < 0.001) and IN tones as less continuous than CN tones (*z* = −13.54, *p* < 0.001). Notably, we also found that the patient group perceived IN tones as more continuous than the control group did (*z* = −2.97, *p* = 0.032). In the patient group, we found no relationships between the continuity ratings of the four conditions and medication, i.e. Chlorpromazine equivalent (IN: *r* = −0.22, *p* = 0.354; IF: *r* = −0.26, *p* = 0.272; CN: *r* = −0.25, *p* = 0.288; CF: *r* = −0.18, *p* = 0.457).

### Electroencephalography

**Figure 2:**
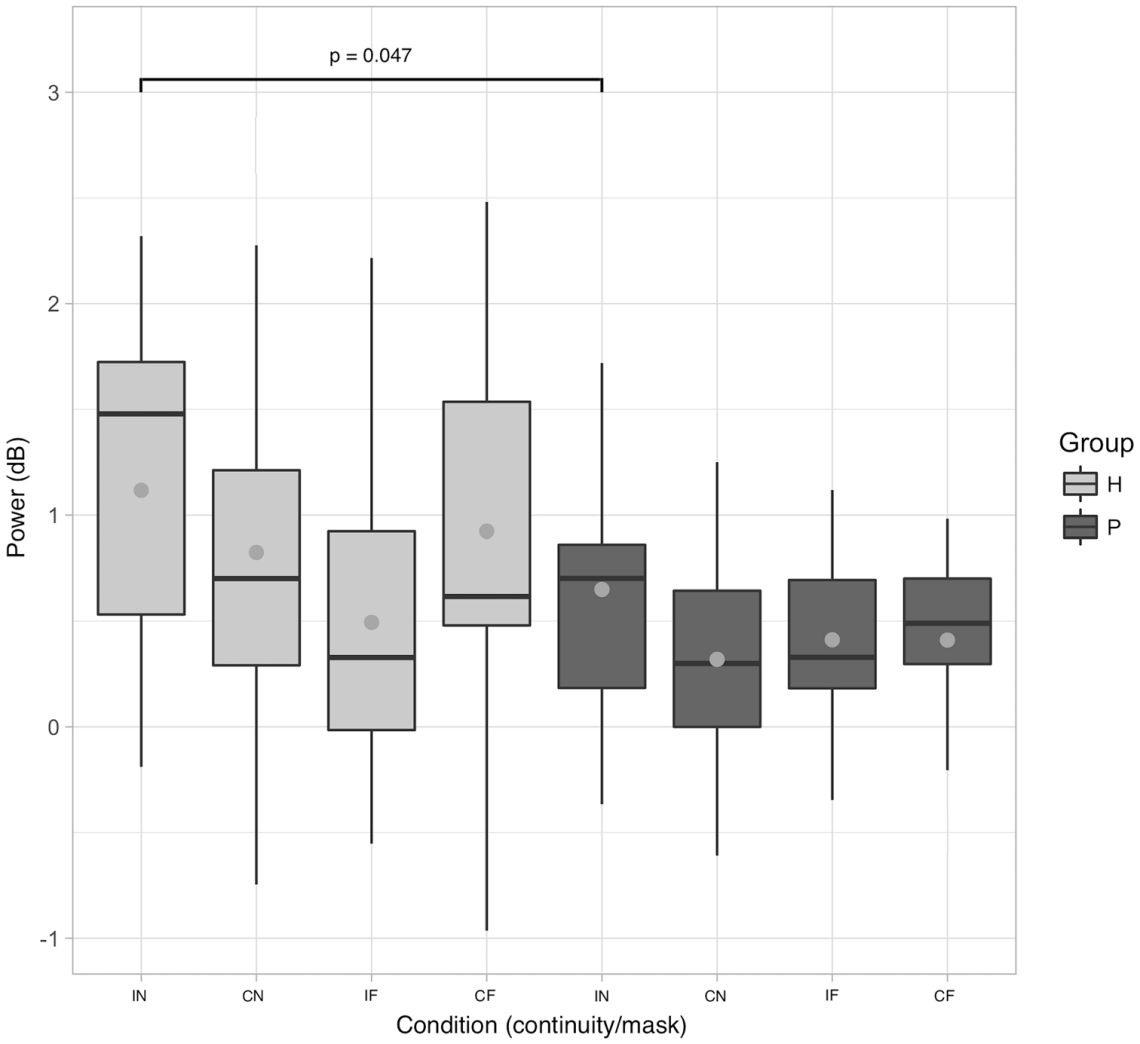
Induced 3 Hz power across conditions. Control data (H) is arranged on the left of the figure (light grey boxes); patient data (P) on the right (dark grey boxes). Grey points indicate mean values. Significance bracket describes the result of an independent samples t-test for the IN healthy versus IN patients contrast.

The ANOVA of induced EEG power at 3 Hz revealed main effects for Mask [*F*(1,105) = 5.91, *p* = 0.017] and Group [*F*(1,35) = 4.33, *p* = 0.045], but not for Tone Continuity [*F*(1,105) = 0.77, *p* = 0.380; Fig. 2].

**Figure 3:**
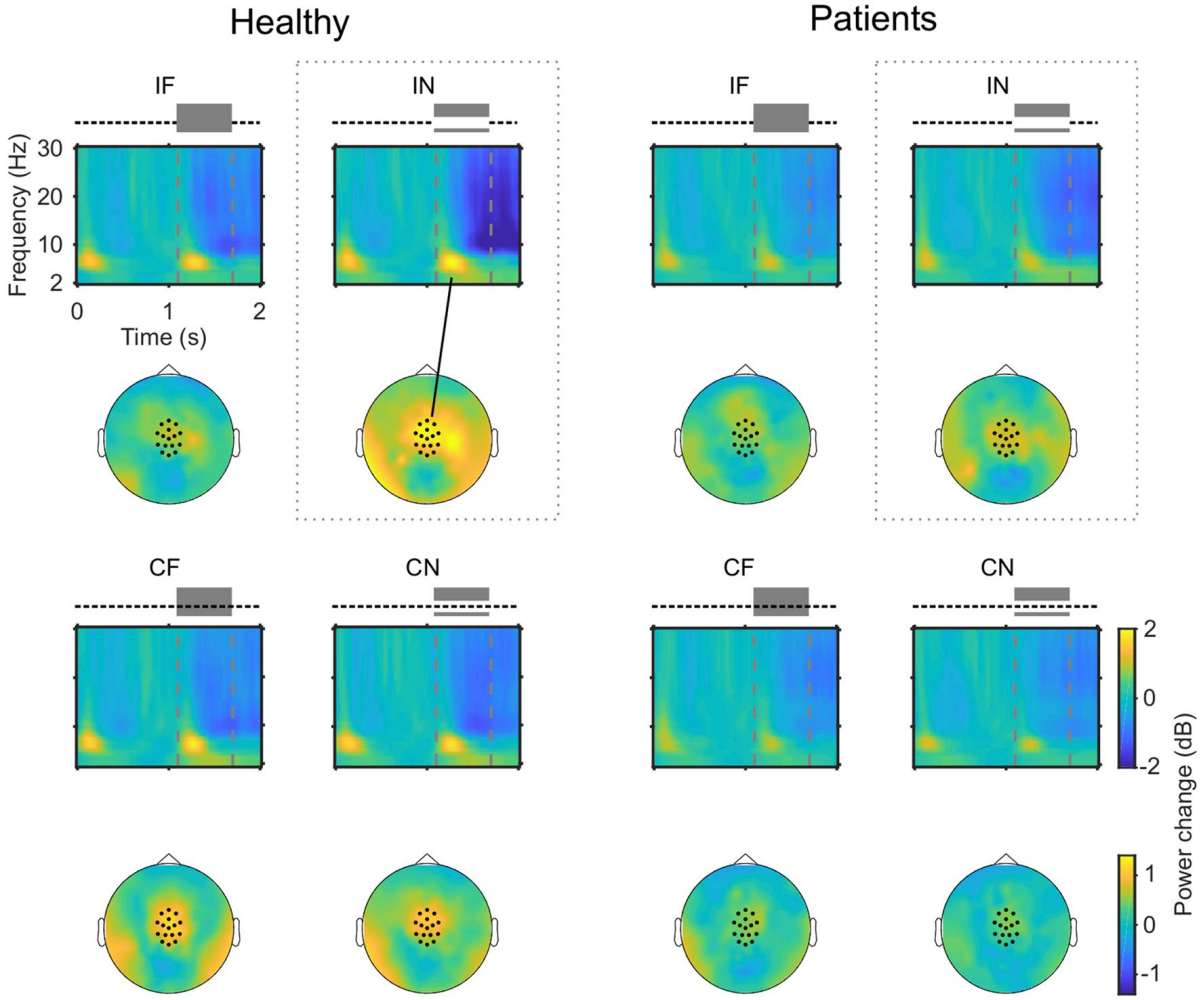
*EEG s*pectrograms and scalp topographies for all conditions. The spectrograms show the power change (relative to baseline), over the electrodes indicated by dots in the scalp maps. Mask onsets and offsets are indicated by dashed grey lines. The topographies show the 3 Hz power change, averaged over the analysed time window (1.2–1.6 s). Mediocentral 3 Hz power in the IN condition is larger in healthy participants compared to patients.

Follow-up comparisons for the main effect of Group revealed reduced 3 Hz power in the patient group across experimental conditions [*t*(35) = 2.082, *p* = 0.045]. Full spectrograms and topographies of 3 Hz power for all groups and conditions are illustrated in Fig. 3. Due to our behavioural finding that the patient group’s continuity rating of the IN condition was higher than that of the control group, we ran an independent samples t-test for induced 3 Hz power for the IN healthy vs. IN patients contrast. This proved to be significant [*t*(35) = 2.06, *p* = 0.047], indicating that induced 3 Hz power was attenuated for the patient group versus the control group in the IN condition.

**Figure 4:**
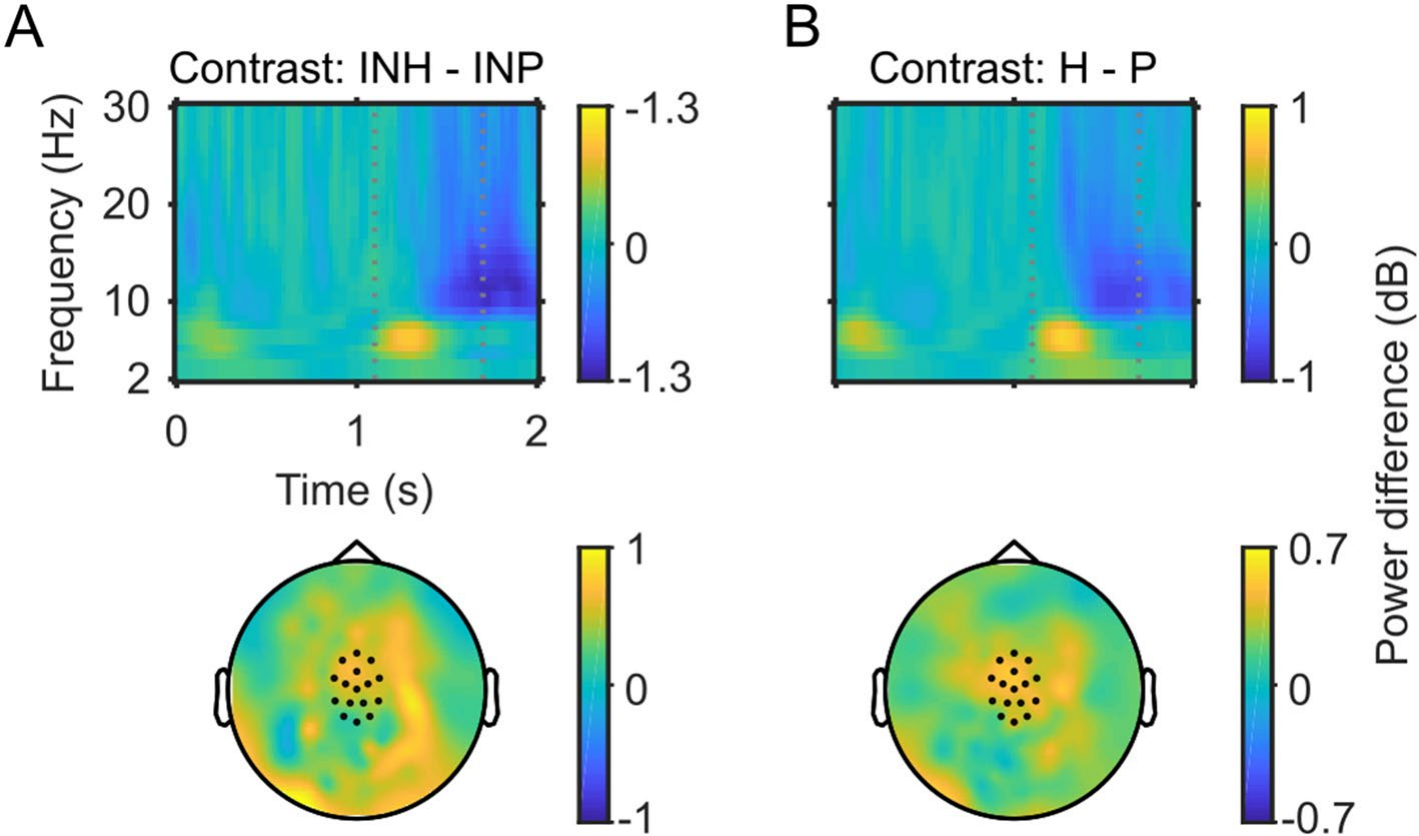
Spectrograms and topographic distributions of contrasts of interest. (**A**) Top row: details a time–frequency plot for the IN healthy controls versus IN patients contrast, indicating increased induced 3 Hz power during the gap/mask in the control group. Noise mask on- and offset are indicated by dashed lines. Bottom row: topographic distribution of induced 3 Hz power change within the time window of interest. Analysed channels are indicated by dots. (**B**) Same as in A, for the contrast between controls and patients pooled across experimental conditions.

**Figure 5:**
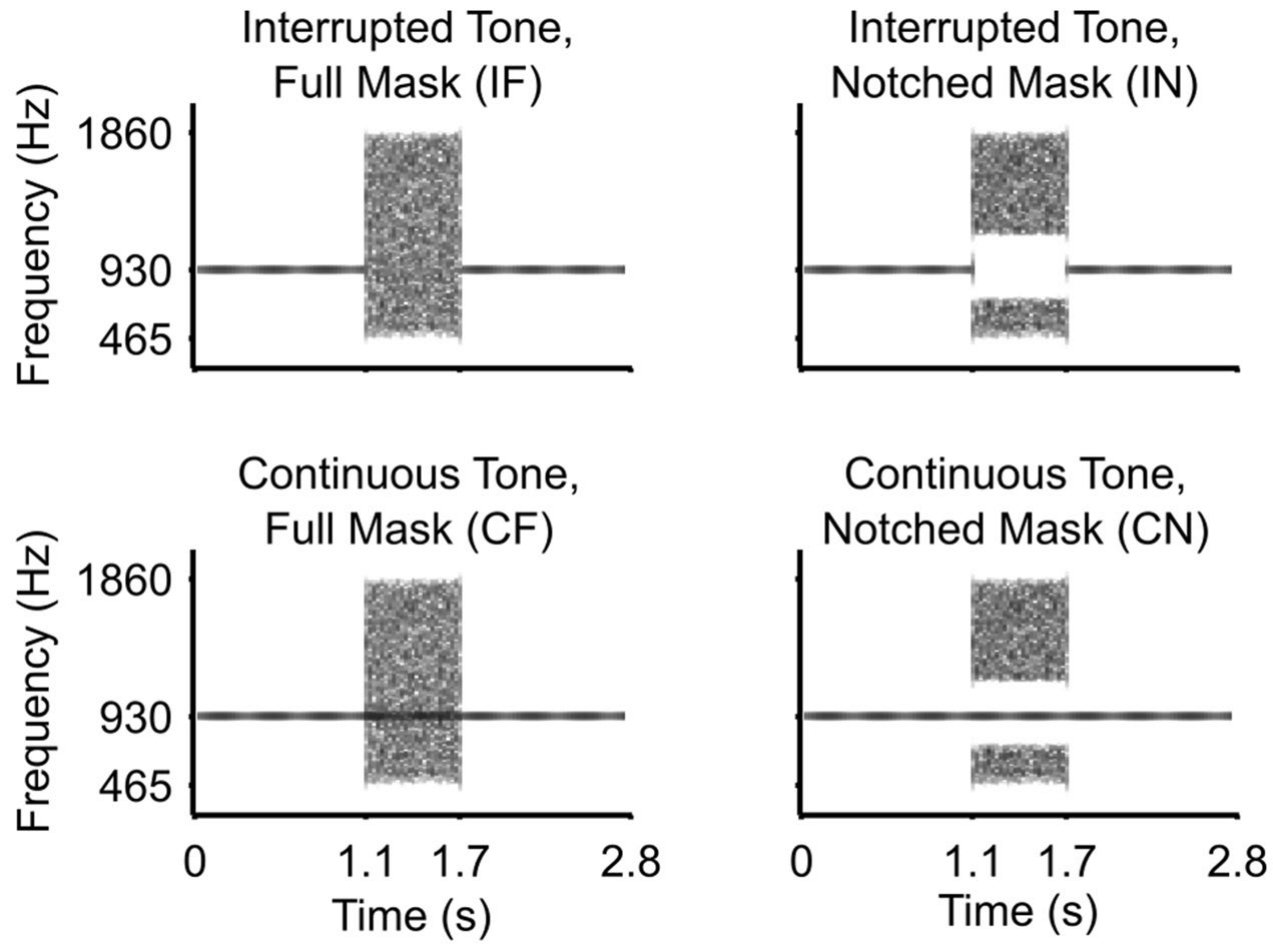
Schematic representation of different stimulus types employed in the experimental paradigm.

Spectrograms and topographic distributions for this contrast, as well the contrast between patients and healthy controls pooled across all experimental conditions, are illustrated in Fig. 4. Next, we compared induced 3 Hz power between patients and controls in the IF condition, as this condition was designed to elicit the continuity illusion. However, an independent samples t-test revealed no difference in induced 3 Hz power between groups in the IF condition [*t*(34) = 0.177, *p* = 0.681]. The spectral extent of the low-frequency power modulation following the mask onset (see Fig. 5) indicates that the low-frequency group effect for the IN condition may be also found in the theta band (4–7 Hz). For exploratory purpose, we also compared theta band oscillations for the IF and IN conditions between groups. This was done for the same time window and mediocentral electrodes for which the 3 Hz effect was found. However, this analysis revealed no differences between groups in either the IN [*t*(35) = 1.911, *p* = 0.643] or IF [*t*(35) = 1.393, *p* = 0.172] conditions. The univariate ANOVA also revealed an interaction between Tone Continuity and Masking Level [*F*(1,105) = 14.98, *p* < 0.001]. This interaction was also found in our previous analyses^15^ and persisted with the addition of the patient data. Follow-up analyses for this interaction were not performed, as we were specifically interested in differences between groups (see “Acquisition and analysis of EEG data” section). Finally, we examined whether medication influences 3 Hz power in the patient group. However, the correlation analyses revealed no relationships between 3 Hz power in the four conditions and the Chlorpromazine equivalent (IN: *r* = 0.03, *p* = 0.893; IF: *r* = −0.09, *p* = 0.686; CN: *r* = 0.35, *p* = 0.126; CF: *r* = 0.11, *p* = 0.637).

## Discussion

In this study we examined continuity ratings and low-frequency oscillations during the ACI in healthy participants and ScZ patients. Compared with healthy participants, ScZ patients showed significantly higher continuity ratings in the interrupted, partially-masked tone condition. This effect was paralleled by significantly reduced 3 Hz oscillations at medio-central electrodes. We found no group differences in behavioural data or EEG data for the other experimental conditions.

Patients rated interrupted, partially-masked tones as more continuous than controls did. The elevated continuity ratings suggest an abnormal bias in patients with ScZ to perceptually fill gaps. A factor that might have contributed to the elevated continuity ratings in the ScZ group is diminished attention capture^15^. Researchers have argued that auditory stream formation depends on selective attention^30^. Moreover, auditory stream formation may influence the ACI by grouping the tone fragments and mask into a single perceptual stream^31^. Hence, it is possible that the enhanced susceptibility of patients with ScZ to perceive the interrupted, partially-masked tones as continuous relates to attention-mediated deficits in auditory stream segregation. In turn, this may result in an abnormally high susceptibility to confuse and group acoustic events from different sound sources, which would explain the elevated continuity ratings in patients. Previous studies have highlighted specific deficits in auditory stream segregation in ScZ patients^32,33^. However, in the present study we did not explicitly measure attention. Therefore, the idea that differences in attention could have contributed to the group differences in continuity ratings remains to be tested.

Patients’ continuity ratings for the other three conditions did not differ from the ratings of the control group. Our finding of high continuity ratings for these conditions is in line with previous research in healthy individuals^20^. Of particular interest is the absence of perception differences between groups in the interrupted, fully-masked condition. Restoration processes in the auditory cortex, which result in a perceptual grouping of tone fragments and a mask, are presumably necessary to perceive these stimuli as continuous^26,34^. Participants from both groups perceived the interrupted, fully-masked tones as continuous. This suggests an intact auditory restoration for these stimuli in patients with ScZ. It is worth noting that stimulus restoration of interrupted, fully-masked tones was almost at ceiling in the control group, so additional enhancement in the ScZ group would not be expected with the current experimental paradigm. Future research could investigate whether stimulus restoration of interrupted, fully-masked tones is also enhanced in patients with ScZ by utilizing ambiguous stimuli with intermediate potential to elicit the ACI (induced, e.g., by longer interruptions). Taken together, our data suggest relatively intact auditory restoration for interrupted, fully-masked auditory stimuli but an enhanced auditory restoration for interrupted, partially-masked tones in ScZ. This latter finding may relate to attention-mediated deficits in auditory stream segregation that could result in an overamplification of auditory restoration.

In both groups, mediocentral 3 Hz power was larger in the interrupted, partially-masked condition, in which the tone was often perceived as discontinuous, compared to those conditions in which the tone was primarily perceived as continuous. This observation is in line with previous reports in healthy individuals^20^ and suggests that the processing and perception of discontinuity is associated with an increase in low-frequency oscillations. Overall, the global pattern of low-frequency power modulations for the different experimental conditions was similar across groups.

However, based on our behavioural findings, we conducted a group comparison of 3 Hz power specifically for interrupted, partially-masked tones. This analysis revealed higher 3 Hz power in healthy controls compared to patients. Thus, the enhanced continuity ratings of patients with ScZ for interrupted, partially-masked tones were paralleled by a reduction of 3 Hz power. Together with the finding of reduced 3 Hz power for the three conditions with high continuity ratings, this suggests that reduced 3 Hz power plays a role in auditory restoration during the ACI. Previously, Riecke and colleagues also found diminished low-frequency power for interrupted, fully-masked tones vs. interrupted, partially-masked tones in healthy individuals^20^. The authors interpreted their finding as a reflection of the perceptual salience of acoustic edges. Thus, the increased continuity ratings of patients with ScZ in the interrupted, partially-masked condition may be indicative of a blurring of perceptual boundaries associated with a deficit in low-frequency oscillations.

Some limitations of the current study should be thoroughly considered. Firstly, we examined a relatively small samples of patients and healthy control participants. This leads to a low statistical power and hence, it is possible that we did not uncover all existing behavioural and EEG differences between healthy individuals and patients with ScZ in the ACI. This issue should be addressed in further studies with larger samples. Second, patients were medicated, which could have influenced the study outcome. Our correlation analyses did not reveal any significant relationships between the current medication status, i.e. the Chlorpromazine equivalent, and behaviour, or 3 Hz power. This suggests that the current medication status does not critically influence behaviour and EEG data in the ACI. Nevertheless, it is possible that long-term medication, as it was the case with most of our patients, contributed to our findings. Therefore, the possible confounding influence of long-term medication should be considered when interpreting the current results. Future studies should investigate non-medicated and ideally first-episode ScZ patients in the ACI.

Our time window of interest was confined to the noise interval, beginning 100 ms after the pre-noise interval (baseline) and lasting for 500 ms. Given that our frequency of interest (3 Hz) has a relatively long period of 333 ms, the time–frequency decomposition inevitably induced some overlap between the two intervals. Consequently, the observed power change may reflect both the noise interval and part of the pre-noise interval. However, this applied equally to all experimental conditions and the auditory stimulus was identical across conditions in the baseline pre-noise interval; thus, partial overlap between the intervals could not confound our main result.

Taken together, our study revealed alterations in auditory restoration during the ACI paradigm both in behavioural as well as in EEG data in patients with ScZ. The enhanced restoration, as reflected in elevated continuity ratings, was associated with an attenuation of low-frequency power. Our findings may result from selective attention deficits in ScZ manifesting in alterations in auditory stream formation and segregation. In summary, our study provides new insight into the perception and processing of the ACI in ScZ. It also adds to a growing body of evidence that aberrant low-frequency oscillations contribute to auditory information processing deficits in ScZ^15,16^.

## Materials and methods

### Participants

Twenty-three patients with ScZ were recruited from outpatient units of the Charité–Universitätsmedizin Berlin. All patients fulfilled the DSM-IV and ICD-10 criteria for ScZ and no other psychiatric axis I disorder. Severity of symptoms in patients with ScZ was assessed with the Positive and Negative Syndrome Scale (PANSS)^35^. Two patients were excluded due to insufficient quality of their EEG data. One additional patient was excluded due to invariant behavioural responses across conditions (see “Analysis of behavioural data” section). The mean age of the remaining 20 participants (14 male, 6 female; 18 right-handed, 2 left-handed) in the patient group was 35 years (range: 23–52 years). Twenty-three healthy control participants matched for age, handedness, education and sex were drawn from the general population. Three control participants were excluded due to insufficient quality of their EEG data. Three additional control participants were excluded from the control group due to invariant behavioural responses across conditions (see “Analysis of behavioural data” section). The mean age of the remaining 17 participants (11 male, 6 female; 15 right-handed, 2 left-handed) in the control group was 36 years (range: 24–50 years). The control group consisted of the same subjects used for the previous analysis^27^. Table 1 provides an overview of demographic data, cognitive performance, and clinical scores of the study participants.

**Table 1:** Overview of demographic data, positive and negative symptoms, and cognitive scores of the study's participants who were included in the final data analysis. Inferential statistics (rightmost columns) refer to differences between patient group and control group. Groups did not differ in terms of age and education, but patients showed deficits in cognitive functions, as assessed by BACS.

	Patients		Controls		Statistics	
	Mean	SD	Mean	SD	*t *value	*p *value
Age (years)	35.15	7.59	36.06	7.96	– 0.61	0.55
Education (years)	11	1.59	11.23	1.68	0.12	0.91
Chlorpromazine Eq. (daily dosage/mg)	337.73	165.23	–	–	–	–

	*N*		*N*			
Gender (m/f)	14/6		11/6		–	–
Handedness (r/l)	18/2		15/2		–	–
Antipsychotic medications	16		–		–	–
Co-medication^a^	4		–		–	–
**BACS (T Score)**						
Verbal memory	44.1	12.47	52.53	10.77	− 2.18	0.04*
Digit	44.05	10.76	49.0	9.42	− 1.47	0.15
Motor	43.5	10.68	50.52	9.81	− 2.07	0.05
Fluency	48.65	9.68	52.18	12.95	− 0.94	0.35
Symbol coding	42.4	9.39	48.53	13.38	− 1.63	0.11
Tower of London	51.05	7.71	53.18	6.15	− 0.89	0.38
Composite T	42.75	8.96	51.53	9.99	− 2.81	0.01*
Total score	249.58	32.79	283.06	33.54	− 2.79	0.01*
**PANSS**						
Positive	16.35	3.59	–	–	–	–
Negative	18.05	5.12	–	–	–	–
General	37.6	6.08	–	–	–	–

^a^Co-medication of antipsychotics and mood-stabilizers.

*p  < 0.05.

All participants had normal hearing (< 25 dB hearing level) and normal or corrected-to-normal vision. None of the healthy participants in the control group had a record of neurological or psychiatric disorders or met criteria for alcohol or substance abuse. To test cognitive performance, the Brief Assessment of Cognition in Schizophrenia (BACS) was assessed^36^. The experimental procedures were conducted in accordance with the 2008 declaration of Helsinki and approved by the ethics committee of the Charité-Universitätsmedizin Berlin (Approval Number: EA1/169/11). Prior to the experiment, each participant gave written informed consent.

### Stimuli and experimental design

The experimental design of the present study was identical to that employed by our previous analyses^27^. The procedures are repeated here for the reader’s convenience.

Auditory stimuli were presented from a central speaker below a cathode ray tube (CRT) monitor and the stimulus intensity was set to 70 dB sound pressure level. During the experiment, a central, dark grey fixation cross was presented on a grey background, which had a luminance of 21 cd/m^2^. Auditory stimuli had a duration of 2.8 s and were sampled at 44,100 Hz. They consisted of 930 Hz pure tones, which were amplitude-modulated at 3 Hz (sinusoidal modulation, depth: 100%, fixed phase across trials), and a noise burst (mask). The tone was either continuous or interrupted with a central 0.6 s silent period. The mask consisted of white noise that was bandpass-filtered into a 2-octave region centred on the tone frequency. The mask started 1.1 s after tone onset, so that it overlapped with the central 0.6 s of the tone. Tone and mask onsets and offsets were linearly ramped with 3 ms rise-fall times, with ramp centres of the mask synchronized with those of the adjoining tones. The masking potential of the noise was manipulated by either bandstop-filtering it in the 0.6-octave range centred on the tone frequency (notched mask), or leaving the noise unchanged (full mask). Hence, the study comprised a 2 × 2 × 2 factorial design with the factors Tone Continuity (continuous vs. interrupted) and Masking Level (notched vs. full), and with the additional between-subject factor of Group (patients vs. healthy controls). We refer to the four stimulus types as continuous tone and full mask (CF); continuous tone and notched mask (CN); interrupted tone and full mask (IF); and interrupted tone and notched mask (IN) (see Fig. 5).

For each of the four stimulus types, 120 trials were presented. These 480 trials were presented across 12 blocks, during which the four stimulus types were randomly intermixed. Participants were asked to rate the continuity of the tone on a scale from 1 (very likely interrupted) to 4 (very likely continuous) by pressing a button with the index finger of the right hand after each trial. The response window started 0.8–1.2 s after tone offset, had a duration of 0.5 s, and was indicated by a red fixation cross. Participants did not receive feedback. The inter-trial-interval varied randomly from 2 to 2.5 s, for a total trial duration of 6.1–7 s. The total duration of the experiment including breaks, but excluding EEG preparation, was approximately 80 min.

### Analysis of behavioural data

To ensure that all participants could distinguish between the four distinct auditory stimuli, a one-way ANOVA with the factor Stimulus Type (IF, IN, CF, CN) was applied to the single-trial behavioural data, i.e. to the continuity ratings. This was done separately for each participant. Only participants who could reliably distinguish between the stimuli, as indicated by a significant (*p* < 0.05) main effect of stimulus type, were included in subsequent analyses. Due to this criterion, four individuals (1 patient; 3 controls) were removed from further analysis.

The further analysis of continuity ratings employed a univariate ANOVA based on a linear mixed effects model with fixed effects of Continuity (interrupted vs. continuous), Mask (full mask vs. notched mask), Group (patients vs. controls), and with Subject as a random effect. Significant effects and interactions were followed up by using multiple-comparison-of-means tests with the ‘multcomp’ package in R^37^, with *p *values corrected for multiple comparisons using the default single-step method. Twelve contrasts were defined in order to test single factor-level comparisons, e.g., IF healthy versus IF patients, or IN patients versus IF patients. Comparisons across different levels of more than one factor, e.g., IN healthy versus CF patients, were not included. Finally, we examined the influence of medication on behavioural performance in the patient group. To this end, Pearson correlation analyses between continuity ratings and medication status, i.e. Chlorpromazine equivalent dosage, were conducted for the four conditions.

### Acquisition and analysis of EEG data

EEG was recorded using an active 128-electrode cap (EasyCap, Herrsching, Germany), including two electrooculography electrodes placed below and lateral to the right eye to monitor eye movements, and Brainamp DC amplifiers (Brainproducts, Gilching, Germany). Data were recorded in reference to an electrode positioned on the nose at a sampling frequency of 1,000 Hz, with an online 0.1 Hz highpass filter and a 250 Hz lowpass filter. EEG data processing was performed in MATLAB (MathWorks, Natick, MA, USA) using the EEGlab^38^ and FieldTrip^39^ toolboxes and custom scripts. Data were high-pass filtered at 1 Hz and low-pass filtered at 150 Hz. The default EEGlab filter settings were applied, resulting in a one-pass non-causal zero-phase Hamming-windowed sync FIR filter, with a filter order of 3,300 and a −6 dB cutoff frequency of 0.5 Hz for the high-pass, and a filter order of 88 and a cutoff frequency of 168.75 Hz for the low-pass filter. Line noise was removed by fitting a 50 Hz sinusoid signal in segments of 4 s and subtracting the fitted signal from the data (*cleanline* plugin in EEGlab). Data were subsequently downsampled to 500 Hz and epoched from −1 to 2.8 s relative to the auditory stimulus onset. Trials and channels that contained large artefacts were removed following visual inspection. Data were subsequently re-referenced to common average and subjected to independent component analysis using the extended infomax algorithm as implemented in EEGlab (*runica*)^40^. Components that represented eye blinks, cardiac, or muscle activity were removed following visual inspection. Subsequently, the electrooculography electrodes were removed from the data and rejected EEG channels were interpolated using spherical interpolation. Trials that still exceeded a threshold of ± 150 µV after these procedures were rejected. For the control group, on average, 43.9 (± 25.7 SD) trials and 10 (± 2.9 SD) ICA components were removed from each dataset. In addition, 6.4 (± 3.5 SD) channels were interpolated. For the patient group, on average, 29.9 (± 17.5 SD) trials and 14.7 (± 4.9 SD) ICA components were removed from each dataset. Moreover, 4.1 (± 3.2 SD) channels were interpolated.

For the analysis of induced neural oscillations, first the condition-specific average waveforms (i.e. event-related potentials) were subtracted from the single trial time series to reduce phase-locked activity. The resulting data were time–frequency transformed using a single Hanning window with a frequency-dependent window length (3 cycles/frequency) at a resolution of 1 Hz. Time–frequency analysis was performed for the interval from − 0.8 to 2.4 s in 25 ms steps. Next, the time–frequency representation was normalized relative to baseline (the 0.5 s interval prior to noise onset, i.e. 0.6–1.1 s after auditory stimulus onset) using decibel conversion. As our previous analyses showed effects around 3 Hz^27^, we also focused the analysis on average 3 Hz power at 16 mediocentral electrodes in the time range of 0.1–0.4 s after noise onset. The region of interest (ROI) and time range was defined based on independent data^20^.

Power of 3 Hz oscillations was analysed using the same statistical model as used for the analysis of behavioural data: a univariate ANOVA based on a linear mixed effects model with fixed effects of Continuity, Mask and Group, and with Subject as a random effect. As we were specifically interested in differences between groups, main effects and interactions concerning the factors Continuity and Mask were not subjected to follow-up analyses in order to avoid circularity. We additionally performed independent samples t-tests contrasting induced 3 Hz power between patients and healthy controls in the IN and IF conditions. Moreover, to examine the influence of medication on 3 Hz oscillations, we computed Pearson correlations between the Chlorpromazine equivalent dosage and 3 Hz power in the four conditions.

## Data Availability

The datasets generated during the current study are available from the corresponding author upon reasonable request.
